# Comparing transplantation sites of mouse immature testicular tissue fragments embedded in chitosan scaffold to maintain tissue integrity: Epididymal fat pad or under the back skin

**DOI:** 10.22038/ijbms.2025.89675.19348

**Published:** 2025

**Authors:** Alireza Anvari, Mansoureh Movahedin, Fariba Ganji

**Affiliations:** 1 Department of Anatomical Sciences, Faculty of Medical Sciences, Tarbiat Modares University, Tehran, Iran; 2 Biomedical Engineering Group, Faculty of Chemical Engineering, Tarbiat Modares University, Tehran, Iran

**Keywords:** Chitosan, Scaffold, Spermatogonia stem cells, Testicular tissue – transplantation, Tissue engineering

## Abstract

**Objective(s):**

Cryopreservation of immature testicular tissue (ITT) is currently the only technique available for restoring fertility in adolescent boys with cancer. Preclinical investigations assessing the development of human transplanted ITT using a nude mouse model have revealed a notable decrease in spermatogonial stem cells.

**Materials and Methods:**

We transplanted ITT from neonatal male NMRI mice (3–5 days old), either embedded in a chitosan (CS) scaffold or without it, into the epididymal fat pad (Epi.fat) and back skin of mature (6–8 weeks old) male NMRI mice. Grafts were recovered 14 days post-transplantation. We evaluated scaffold cytotoxicity, morphology, *in vivo* degradation, tissue integrity, seminiferous tubule morphometry, and germ cell survival.

**Results:**

The diameters of the seminiferous tubules did not differ significantly between grafted groups. However, the height of the seminiferous epithelium in the Epi.fat + S group was significantly greater than in the Back + S group. The total seminiferous tubule area ratio in the Epi.fat, Epi.fat + S, and Back groups were significantly lower compared to the Back + S group. The number of PLZF-positive cells per seminiferous tubule section was significantly higher in the Back + S group than in the Epi.fat group.

**Conclusion:**

Allotransplantation of ITT embedded in a CS scaffold enhanced spermatogonial survival and tissue integrity in both the Epi.fat and subcutaneous back skin. Among the two transplantation sites, the subcutaneous back skin yielded more favorable outcomes.

## Introduction

Immature testicular tissue (ITT) remains the only clinically feasible option for fertility preservation in prepubertal boys undergoing gonadotoxic treatments. While experimental approaches, such as spermatogonial stem cell (SSC) transplantation, *in vitro* spermatogenesis, and testicular tissue grafting (1), are under development, none have yet reached clinical application. Cryopreservation of ITT has been proposed as a strategy for long-term fertility preservation in patients undergoing gonadotoxic treatments. However, significant obstacles remain before this becomes a reality in fertility restoration in clinical practice. Human SSC transplantation is limited because of the lack of recognized methods for proliferating SSCs *in vitro* and the inadequate co-transplant environment required for their maintenance (2). Testicular tissue pieces can be implanted into the host as an alternative to SSC suspensions, preserving the interaction between germ and somatic cells by maintaining the SSCs in their native microenvironment (3). When ITT from various animals was transplanted orthotopically or heterotopically into immunodeficient mice, tissue maturation and full spermatogenesis occurred (4-9). After grafting human ITT into mice, the stem cells were observed to differentiate up to the pachytene spermatocyte stage (10-16). However, the generation of mature sperm from transplanted human ITT has not yet been achieved. The primary obstacles in human ITT include reperfusion loss and hypoxia, as well as the maintenance of early spermatogonial populations and the inadequate or delayed development of neovascularization in the transplanted testicular tissue (17). The choice of transplantation site plays a critical role in graft survival and function. Testicular tissue from various animals has been transplanted into the dorsal skin of mice, resulting in successful sperm production (5, 8, 18-20). Nevertheless, human ITT xenografts in dorsal skin, intratesticular, and the scrotum have not achieved complete spermatogenesis, often halting at the pachytene spermatocyte stage (21). Ectopic sites, like the skin, provide ideal locations for gametogenesis (7). Furthermore, the subcutaneous layer of the skin has a significant concentration of capillaries (22). The epididymal fat pad (Epi.fat) plays a crucial role in the process of spermatogenesis. Spermatogenesis stopped after Epi.fat removal (23, 24). The Epi.fat may secrete a specific substance that promotes spermatogenesis by directly affecting the nearby testis (23). Additionally, compared to the ectopic (back skin) site, the orthotopic (scrotum) site has optimum temperatures for spermatogenesis (25). Research on the production of bioengineered scaffolds capable of supporting testicular cells and enhancing ITT transplantation in numerous animals suggests that this approach has promise for preserving human fertility (21). Chitosan (CS), a natural polymer derived from chitin, has shown promise due to its biodegradability, biocompatibility, and ability to form porous structures that facilitate nutrient and oxygen exchange (26, 27). CS-based scaffolds have shown rapid degeneration, preserving normal cell morphology, increasing viability, good proliferation, and attachment of stem and living tissue cells (28). Naeemi et al. demonstrated that CS-based scaffolds containing decellularized testicular tissue and hyaluronic acid could adequately function as a supportive layer for the differentiation and proliferation of SSCs (29).

This study introduces a novel comparative transplantation approach by evaluating two anatomically distinct sites—the epididymal fat pad and the dorsal skin—for preserving ITT using a chitosan-based scaffold. Unlike previous studies that primarily focused on a single site, our dual-site design provides new insights into the role of local tissue environments in supporting graft integrity. The main goal of this research was to produce a novel scaffold based on biodegradable, biocompatible, accessible, and natural materials with an easy production process suitable for testicular tissue graft. We also compared two potential transplantation sites, the back skin and the epididymal fat pad, for improved transplant results.

## Materials and Methods

### Animals

The experimental group consisted of 8 newborns (3–5 days old) male NMRI mice as donors and 16 mature (6–8 weeks old) male NMRI mice as recipients. Four male mice (3 weeks old) were used as the control. The mice were sourced from the Pasteur Institute of Iran. In the colony room, animals were housed and bred with access to food and water under a controlled 12-hr light/dark cycle. Animals were randomly allocated to experimental groups using a random number generator, and the allocation sequence was concealed until interventions were assigned. All surgical procedures and assessments were conducted by ethical standards approved by Tarbiat Modares University (Permission No. IR.MODARES.AEC.1401.001). To minimize observer bias, outcome assessments were performed in a blinded fashion—investigators analyzing histological and fluorescence data were unaware of the group assignments. No adverse animal events were observed during the study. These procedures comply with the ARRIVE 2.0 guidelines.

### Preparation of the Chitosan scaffold

The CS scaffold preparation technique used in this study was previously published elsewhere (30). In summary, for preparing 10 ml of CS solution (2 w/v %) (Mw =190,000–310,000 Da; 75–85 % degree of deacetylation, viscosity 200–800 cP, Sigma, USA), 200 mg of CS powder was dissolved in 2 ml of distilled water and 6 ml of acetic acid (0.1 M), followed by vigorous stirring for 24 hr at room temperature. Additionally, 600 mg of β-glycerol phosphate (β-GP) (Merck, Germany) was dissolved in 2 ml of distilled water. The CS solution was then stirred continuously at 4 °C while the β-GP solution was added dropwise. The final pH of the CS/β-GP solution was measured to be approximately 7.2, which is suitable for gelation and cell compatibility. The scaffolds were molded and incubated for fifteen minutes at 37 °C, then frozen at –80 °C and freeze-dried. Before transplantation, the scaffolds were cut to roughly 4 mm in diameter and 2 mm in height. They were sterilized using 70% ethanol and ultraviolet light for 1 hour. Although no microbial culture testing was performed, sterility was assumed based on the combined UV and ethanol treatment, which is commonly used in scaffold preparation protocols (31). Finally, the scaffolds were thoroughly washed with sterile phosphate-buffered saline (PBS) and incubated in culture medium at 37 °C for two hours before use.

### Morphological evaluations of the scaffold

The morphology of the synthesized scaffold was studied using scanning electron microscopy (SEM; KYKY Technology Development, Beijing, China). In preparation for SEM imaging, the scaffold was fixed for four hours at 4 °C in 2% glutaraldehyde (Sigma, USA). The specimen then underwent a series of ethanol dehydration processes (30, 50, 70, 90, and 100%) and was dried using a freeze-dryer. After sputter-coating with a thin layer of gold, the sample was examined under SEM.

### In vivo degradation

To evaluate the *in vivo* degradation properties of CS scaffolds, sterilized scaffolds were subcutaneously implanted into the dorsal region of NMRI mice. Anesthesia was induced via intraperitoneal injection of ketamine (80 mg/kg) and xylazine (10 mg/kg), both dissolved in PBS (Alfasan, Netherlands). Following implantation, the skin incision was closed using 4-0 nylon sutures under sterile conditions. Postoperative analgesia was provided by administering buprenorphine (0.1 mg/kg, subcutaneously) immediately after surgery and again 24 hr later to minimize discomfort. Mice were sacrificed at predetermined timepoints— 0, 7, 14, 20, 25, and 30 days post-implantation—and the scaffold implantation sites were carefully dissected.

### Cytotoxicity assay

To assess the scaffolds’ cytotoxicity and cell viability, the 3-[4,5-dimethyl(thiazol-2yl)-3,5-diphenyl] tetrazolium bromide (MTT, Carl Roth, Germany) assay was used. SSCs from neonatal NMRI mice (3–5 days old), obtained from the Pasteur Institute of Iran, were seeded onto the scaffolds. The scaffolds were sectioned into 2 × 2 × 2 mm³ pieces and placed in a 96-well plate. They were sterilized with 70% ethanol and ultraviolet radiation for one hour, then thoroughly washed with sterile PBS. Afterwards, the scaffolds were incubated in complete culture medium at 37 °C for two hours to precondition and equilibrate before cell seeding, which was sufficient to achieve equilibrium. A total of 3 × 10³ SSC were seeded in each well onto the scaffolds and cultured in Dulbecco’s Modified Eagle Medium (DMEM, Gibco, USA) supplemented with 10% fetal bovine serum (FBS, Gibco, Germany) for 72 hr. The MTT assay was performed at 24, 48, and 72 hr post-seeding. To assess formazan formation, 200 μl of culture medium containing MTT (0.5 mg/ml) was added to each well and incubated at 34 °C for four hours. After removing the medium, the resulting formazan crystals were dissolved in dimethyl sulfoxide (DMSO; Carl Roth, Germany). The optical density (OD) of the supernatant was measured at 570 nm using a microplate reader (Beckman, Fullerton, CA, USA). The control group consisted of cells cultured in medium on plastic plates. For each experimental group, three biological replicates were prepared. Each replicate was seeded in a 96-well plate and treated according to the experimental conditions (29).

### Preparation of ITT

Mice were euthanized by brief exposure (<5 minutes) to carbon dioxide, followed by cervical dislocation to ensure complete sacrifice. The testes of the donor mice (3–5 days old) were obtained and rapidly placed in ice-cold PBS. The testes were dissected into small pieces, each measuring approximately 1 mm³. DMEM with a 10% FBS supplement kept testis fragments on ice (32).

### Experimental design

Testicular tissue fragments were assigned to four experimental groups (n = 4 mice per group):

1. Epi.fat: Transplantation into the epididymal fat pad

2. Epi.fat + S: Transplantation into the epididymal fat pad with CS scaffold

3. Back: Transplantation into the dorsal subcutaneous skin

4. Back + S: Transplantation into the dorsal skin with CS scaffold

Fragments from 3-week-old mice served as ungrafted controls. Group assignments were randomized using a random number generator, and allocation was blinded to outcome assessors to reduce bias.

### Transplantation of testicular tissue fragments

To induce anesthesia, recipient NMRI mice received an intraperitoneal injection of ketamine (80 mg/kg) and xylazine (10 mg/kg). Following surgical preparation under aseptic conditions, bilateral castration was performed via scrotal incisions immediately before testicular tissue transplantation (7). The mice were divided into four experimental groups:

• Groups 1 and 3 received two fragments of testicular tissue transplanted into the Epi.fat and dorsal skin regions, respectively.

• Groups 2 and 4 received two fragments of testicular tissue embedded in CS scaffolds, transplanted into the Epi.fat and dorsal skin regions, respectively.

Postoperative analgesia was provided by administering buprenorphine (0.1 mg/kg, subcutaneously) on the day of surgery and again 24 hr later to minimize pain and ensure animal welfare.

### Graft recovery

After two weeks, the recipient mice were sacrificed by cervical dislocation. The graft region was meticulously dissected. The number of transplanted tissues was recorded and isolated, then fixed in 4% paraformaldehyde (for immunofluorescent analysis) or Bouin’s solution (75 ml saturated aqueous picric acid, 25 ml formalin, and 5 ml glacial acetic acid) for morphometric analysis.

### Histology and immunofluorescent analysis

*Histology*

Following fixation and standard histological processing, tissues were incorporated in paraffin and then cut into serial sections with a thickness of 5 μm. For histological assessment, hematoxylin and eosin (H&E) staining was applied to one section every 25 μm. 

For scoring, we examined 438 seminiferous tubule sections, with a ratio of 1–1.5 between the two perpendicular diameters of the tubules. These sections were categorized into five groups: 125 for Epi.fat, 125 for back skin, 80 for Epi.fat with scaffold, and 108 for back skin with scaffold. For morphometry analysis, 100 seminiferous tubule sections from 3-week-old mice were added as the control group. The tubules were assigned a numerical score from 1 to 3 based on the following criteria: score 1 for seminiferous tubules with cell cohesion, cell adhesion to the basement membrane, and lack of sclerosis (intact); score 2 for tubules showing clearly identified intratubular cells despite localized necrosis (satisfactory); and score 3 for tubules with complete necrosis (damage) (33) (Figure 4 A). The following parameters were manually measured using Motic software: tubular diameter (μm), height of the seminiferous epithelium (μm), and the area ratio of seminiferous tubules (34). To assess structural changes in the testicular tissue, the area ratio was calculated using the following formula:

Area ratio (%) = (Total seminiferous tubule area / Total tissue cross-section area) × 100

*Immunofluorescent*

The whole-mount immunofluorescence technique was applied to seminiferous tubules following the previously established protocol (35). After fixation and permeabilization, samples were blocked with 2% bovine serum albumin (BSA) for one hour at room temperature to prevent nonspecific binding. The tubules were then incubated overnight at 4 °C with a primary antibody against PLZF (1:50, sc-28319, Santa Cruz Biotechnology), a marker of undifferentiated spermatogonia. After thorough washing with PBS, samples were incubated for one hour at room temperature with Alexa Fluor-conjugated secondary antibodies: Alexa Fluor 488 (A-11008) or Alexa Fluor 546 (A-11030) (Thermo Fisher Scientific), diluted according to the manufacturer’s instructions. Nuclei were counterstained with DAPI (4′,6-diamidino-2-phenylindole) for 10 minutes at room temperature. Samples were then mounted with fluorescence mounting medium and visualized under a fluorescence microscope. Image acquisition and analysis were conducted using ImageJ software. Negative controls (omission of primary antibody and isotype controls) were included to confirm staining specificity. No detectable fluorescence signal was observed in these controls.

### Statistical analysis

All statistical analyses were conducted using GraphPad Prism 9 (GraphPad Software, La Jolla, CA, USA). The normality of data distribution was tested with the Shapiro-Wilk test. For parametric data (including tissue integrity, seminiferous epithelium height, total seminiferous tubule area ratio, and MTT cell viability), one-way ANOVA followed by Tukey’s *post hoc* test was used. Non-parametric data (seminiferous tubule diameter and immunofluorescence results) were analyzed with the Kruskal-Wallis test and Dunn’s *post hoc* correction. To evaluate the adequacy of the sample size (n = 3 per group for PLZF⁺ cell quantification), an a priori power analysis was performed using GPower software. Based on an estimated effect size from pilot data and a significance level of α = 0.05, the current sample size was adequate to achieve a statistical power of 0.80 for detecting differences in primary outcome measures. All data are presented as mean ± standard deviation (SD), and *P*-values < 0.05 were considered statistically significant.

## Results

### SEM observations

**Figure 1:**
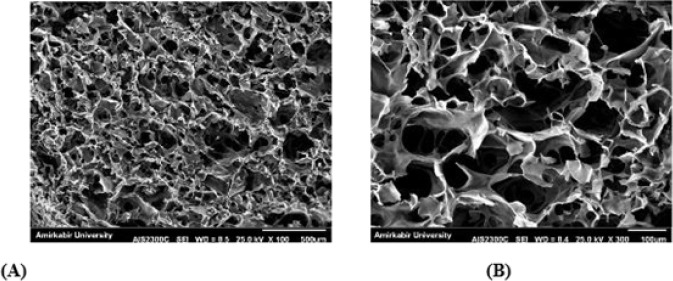
Scanning electron microscopy (SEM) images of the CS scaffold reveal a three-dimensional, multi-porous architecture with interconnected pores (A) Low magnification view at 100× showing overall surface topology. (B) Higher magnification at 300× highlights pore distribution and interconnectivity. CS: chitosan

Scanning electron microscopy (SEM) images of the CS scaffold showed a highly porous, three-dimensional structure with interconnected pores (Figure 1A, B). The scaffold’s morphology was observed at magnifications of 100× and 300×, respectively, confirming its suitability for cell infiltration and nutrient diffusion.

### In vivo degradation

**Figure 2:**
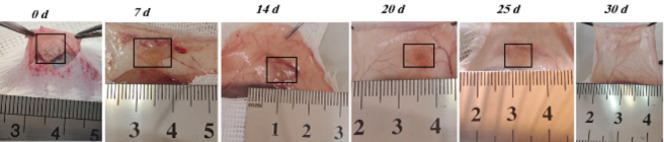
Time-course of *in vivo* degradation of the CS scaffold following implantation

To evaluate *in vivo* degradation, CS scaffolds were subcutaneously implanted into the dorsal region of mice (n = 4). Progressive degradation was observed over time (Figure 2). By day 14, a significant portion of the scaffold had resorbed, and complete degradation was evident by day 30, indicating excellent biodegradability.

### MTT cell viability assay

**Figure 3:**
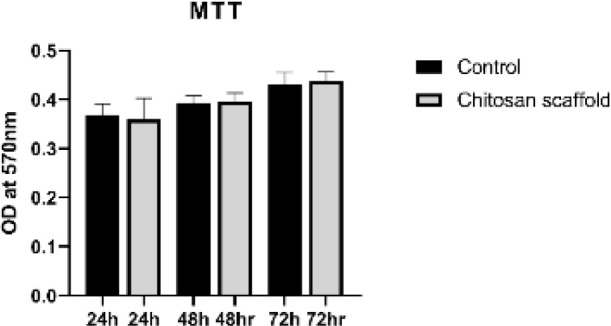
Cytotoxicity of the CS scaffold was assessed using the MTT assay following SSC culture at 24, 48, and 72 hr

The cytocompatibility of CS scaffolds was assessed using the MTT assay on SSC (n = 3). As shown in Figure 3, no statistically significant differences in cell viability were observed between the scaffold and control groups at 24, 48, and 72 hr, indicating that the scaffold did not exert cytotoxic effects.

### Tissue integrity

**Figure 4:**
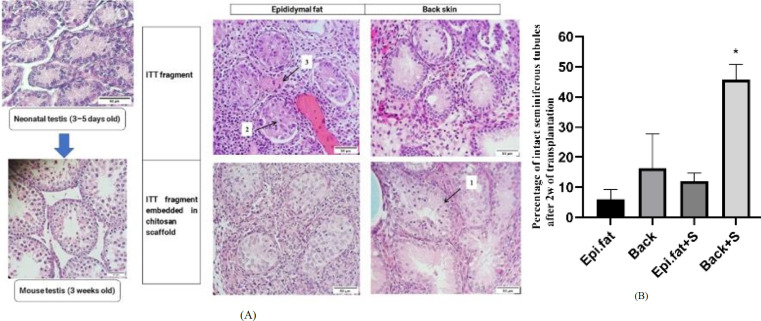
A, B. Evaluation of seminiferous tubule integrity following transplantation of ITT with CS scaffolds into two anatomical sites: Epi.fat and subcutaneous back skin, assessed after 2 weeks. (A) Representative H&E-stained sections showing seminiferous tubules categorized into three integrity scores: Score 1 (intact), Score 2 (satisfactory), and Score 3 (damaged). (B) Quantitative analysis of the percentage of intact seminiferous tubules (Score 1) in each group.

To assess the impact of CS scaffolds and transplantation sites on ITT integrity, grafts were placed in the Epi.fat or under the back skin (n = 4 per group). After 2 weeks, H&E staining was used to evaluate seminiferous tubule integrity (Figure 4A).

 As Table 1 shows, the score 1 of the Back + S group was significantly higher than that of the other groups. No statistically significant difference was observed among the four groups for score 2. The score of 3 in the Epi.fat group was higher than in the other groups, which was a significant difference compared to the Epi.fat + S and Back + S groups.

The results showed that the CS scaffold and back skin graft site demonstrated the highest efficacy in maintaining the integrity of the seminiferous tubule (Figure 4B).

### Morphometry

As presented in Table 2, seminiferous tubule diameters were significantly reduced in all grafted groups compared to the control group, with no statistically significant differences observed among the grafted groups. The height of the seminiferous epithelium was also significantly diminished in all grafted groups relative to the control. However, epithelial height in the Epi.fat and Epi.fat + S groups was significantly greater than in the Back group, and the Epi.fat + S group exhibited a markedly higher epithelial thickness compared to Back + S. Additionally, the total seminiferous tubule area ratio was significantly lower in all grafted groups compared to the control. Among the grafted groups, Epi.fat, Epi.fat + S, and Back demonstrated significantly reduced area ratios in comparison to Back + S.

### Spermatogonial survival

**Figure 5:**
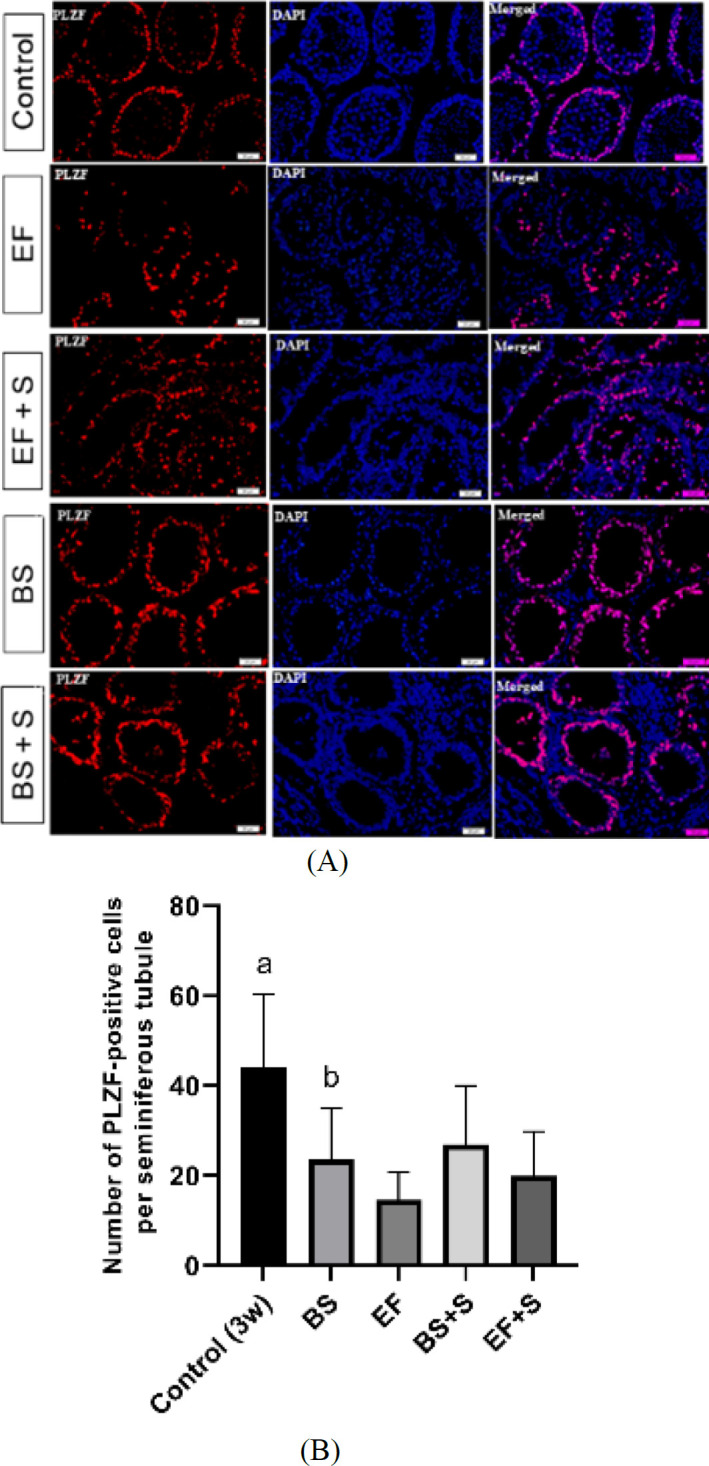
A, B: Evaluation of spermatogonial stem cell (SSC) survival following transplantation of mouse testicular tissue onto chitosan scaffolds at different anatomical sites, assessed two weeks post-transplantation

To quantify SSCs, we employed immunofluorescence against promyelocytic leukemia zinc finger (PLZF) in transplants (n = 3). (Figure 5A). After 14 days, the number of PLZF-positive cells per seminiferous tubule section in all grafted groups was significantly lower than in the control group, and the Back + S group was significantly higher than the Epi.fat group (Figure 5B).

## Discussion

Autologous testicular tissue transplantation (ITT) is a promising strategy for restoring fertility in prepubertal males undergoing gonadotoxic treatments. Initially demonstrated in mice, this approach has shown that sperm derived from both fresh and cryopreserved grafts can fertilize oocytes and produce viable offspring (4, 5, 7). According to experiments involving the transplantation of human ITT into other species, it was observed that the number of spermatogonia decreased the most within the initial three weeks after transplantation. This period is critical for establishing stable vascularization (36). All procedures designed to restore a patient’s fertility using his own frozen-thawed ITT, either *in vitro* or *in vivo*, depend on the existence of spermatogonia as precursors of sperm (37). Thus, improving spermatogonial survival in cryo-stored ITT is a problem with the fertility restoration method (38). 

A distinguishing feature of our study is the integrated evaluation of both scaffold material and anatomical graft site, revealing a synergistic effect between the CS scaffold and dorsal skin location. This dual-variable approach provides new mechanistic insight into how microenvironmental factors interact with biomaterials to influence graft success. CS was selected due to its known biocompatibility, anti-inflammatory properties, and ability to support tissue regeneration and angiogenesis (39), making it a promising scaffold for testicular tissue engineering. 

To address the challenge of spermatogonial survival, we designed a CS scaffold that promotes tissue viability and assessed how the anatomical location of the graft affects transplant success. We showed that incorporating testicular tissue fragments in the CS scaffold, followed by transplantation into the Epi.fat and under the back skin region, significantly enhanced spermatogonial survival and tissue integrity. The superior performance of the back skin site may be attributed to enhanced vascularization, reduced inflammation, and improved oxygenation. However, these hypotheses require validation through targeted mechanistic studies, including vascular staining (e.g., CD31, VEGF), temperature mapping, and cytokine profiling.

To validate transplant success, we assessed seminiferous tubule morphology, structural integrity, and SSC (PLZF⁺) survival at 14 days post-grafting. This time point was strategically selected to evaluate early vascular integration and the onset of histological maturation, while minimizing the confounding effects of tissue remodeling and scaffold degradation. Our choice reflects a compromise between acute-phase assessments (e.g., day 5) and later evaluations (e.g., day 21), as reported in previous studies (36, 40). Although mature sperm formation was not evaluated due to logistical constraints, prior work has demonstrated that early-stage indicators—such as the presence of PLZF⁺ spermatogonial stem cells and intact seminiferous architecture—are predictive of subsequent spermatogenic progression (36, 41). Thus, our findings support the potential for fertility restoration, albeit without direct functional confirmation. Morphometric analysis showed reduced tubular diameter, epithelial height, and seminiferous tubule area ratio in all grafted groups compared to controls. No significant differences were found among grafted groups in tubular diameter, although large standard deviations were noted. Given the small sample size (n = 3), formal outlier analysis was limited; however, no extreme values were visually apparent. The observed variability likely reflects biological differences in graft responses across anatomical sites rather than measurement error. In the Back and Back + S groups, reduced epithelial height may be due to lumen formation and cellular adhesion to the basement membrane. The Epi.fat may favor epithelial maturation within individual tubules, while the dorsal skin site better supports overall tubule formation and organization. This highlights the complexity of recreating the testicular niche and the need to balance multiple developmental parameters.

When comparing the outcomes of the two transplant sites, the back skin region showed superior results. The increased PLZF+ cell count observed in the Back + S group suggests that this transplantation site better supports spermatogonial stem cell niches, potentially via improved oxygenation and scaffold biocompatibility. Transplanting ITT to orthotopic or ectopic sites has shown variable outcomes across species. For example, monkey ITT transplanted into the scrotal region successfully completed spermatogenesis, while ectopic grafts under the back skin arrested at the meiotic level (42). These differences highlight the importance of local microenvironmental factors in graft maturation.

The subcutaneous layer of the skin has a dense network of capillaries that may facilitate rapid revascularization and reduce hypoxia post-transplantation. Prior research has shown that Injury-induced angiogenesis improves graft viability, as demonstrated in ovarian tissue transplantation (42, 43). Our integrated approach reveals location-dependent effects of the scaffold, offering new biological insights into how microenvironmental factors contribute to tissue regeneration. This study is the first to systematically assess how scaffold performance varies across anatomical sites, offering novel insight into location-dependent biomaterial behavior. 

The back skin site, when coupled with the CS scaffold, exhibited superior structural preservation and stem cell maintenance (indicated by PLZF+ cell counts and tubule integrity), which was not observed in other combinations. This novel synergy suggests a scaffold–site dependent mechanism that may involve improved angiogenesis, reduced inflammation, or enhanced ECM remodeling. Our findings reveal a novel interplay between scaffold composition and graft location, demonstrating that CS scaffolds interact differently with distinct tissue environments, influencing structural preservation. This study suggests that the dorsal skin site promotes superior maintenance of seminiferous tubule integrity, potentially due to its more robust vascular supply compared to the Epi.fat. These observations suggest that not only the scaffold material but also its anatomical context plays a mechanistic role in graft success, contributing new insights into site-dependent scaffold performance.

### Limitations

This study has several limitations that should be acknowledged. First, the short observation period (14 days) might not fully represent long-term spermatogenic progression or functional fertility outcomes, such as sperm production and restoration of reproductive capacity. Second, the immunohistochemical analysis was limited to early germ cell markers and did not include more advanced differentiation markers such as SYCP3 or CREM, which restricts the interpretation of later stages of germ cell development. Third, vascularization was not comprehensively assessed; future studies should incorporate angiogenic markers such as CD31 and VEGF to validate site-specific differences in graft integration. Finally, the relatively small sample size per group (n = 3–4), although consistent with previous studies (36), may have reduced statistical power. Increasing the number of biological replicates could enhance the robustness of the findings. Nonetheless, no visual outliers were detected, and the observed variability was attributed to inherent biological heterogeneity across graft sites.

### Future directions

Future research should focus on investigating long-term graft viability, complete spermatogenic progression, and the functional potential of the generated sperm. Building upon the current findings, a follow-up study has been conducted to assess spermatogenesis over extended post-transplantation intervals, and its results will be presented in a separate manuscript currently in preparation. To further optimize graft performance and better replicate physiological conditions, future studies may incorporate exogenous hormonal stimulation and temperature monitoring. Moreover, functional fertility assessments—including sperm retrieval and evaluation of fertilization capability—will be essential for advancing the clinical translation of this approach.

Representative images at 0, 7, 14, 20, 25, and 30 days post-implantation show the progressive resorption of the scaffold and its integration into the host tissue. The scaffold gradually loses its structural integrity, indicating ongoing biodegradation. CS: chitosan

Data are presented as mean ± SD (n = 3). No significant cytotoxic effects were observed compared to the control group, indicating biocompatibility of the scaffold over the tested time points

CS: Chitosan, SSC: Spermatogonial stem cell

Indicates a statistically significant difference compared to other groups (*P*<0.05, n = 4). CS: chitosan, SSC: spermatogonial stem cell, Epi.fat: epididymal fat pad, PLZF: promyelocytic leukemia zinc finger; CS: Chitosan; ITT: Immature testicular tissue; Epi.fat: Epididymal fat pad

**Table 1:** Effect of a chitosan scaffold and transplantation site on seminiferous tubule integrity in mouse testicular tissue after two weeks

	Epi.fat	**Epi.fat + S**	**Back**	**Back + S**
**Score1** **Score2** **Score3**	6% ± 3.26 47.78% ± 19.03 46.23% ± 20.29	12% ± 2.68 82.25% ± 4.41 2.75% ± 1.87^b^	16.30% ± 11.38 54.13% ± 34.89 29.57% ± 23.9	45.67% ± 5.05^a^ 52.30% ± 5.2 2.03% ± 1.84^b^

Tissue integrity was evaluated based on the percentage of seminiferous tubules assigned to each integrity score, and results are presented as mean ± SD (n = 4). Statistical analysis was performed to compare groups within each scoring category. a: Indicates a significant difference compared to other groups in the same row. b: Indicates a significant difference compared to the Epi.fat group in the same row. Differences were considered statistically significant at *P*<0.05.

**Table 2:** Quantitative analysis of seminiferous tubule diameter, epithelium height, and area ratio in mouse testicular tissue following transplantation onto chitosan scaffolds at different anatomical sites

Groups	**Diameter of ** **seminiferous tubules (μm)**	**Seminiferous epithelium height** **(μm)**	**Total seminiferous tubule area ratio (%)**
**Control (3w)** **Epi.fat** **Epi.fat + S** **Back** **Back + S**	145.4 ± 25.15^a^ 79.32 ± 19.98 86.97 ± 19.06 83.07 ± 26.17 82.82 ± 18.75	48.43 ± 12.34^a^ 38.69 ± 9.31 42.2 ± 9.79 30.36 ± 9.74^b^ 34.73 ± 10.04^c^	89.08 ± 1.35^a^ 33.65 ± 8.38 43.3 ± 12.64 38.43% ± 13.98 66% ± 7.68^d^

Values are presented as mean ± standard deviation for each experimental group (n = 4). Statistical comparisons were made within each column. a: Significant difference compared to all other groups in the same column. b: Significant difference compared to Epi.fat and Epi.fat + S groups in the same column. c: Significant difference compared to the Epi.fat + S group in the same column. d: Significant difference compared to Epi.fat, Epi.fat + S, and Back groups in the same column. Differences were considered statistically significant at *P*<0.05. Epi.fat: Transplantation into the epididymal fat pad; Epi.fat + S: Transplantation into the epididymal fat pad with CS; scaffold Back: Transplantation into the dorsal subcutaneous skin; Back + S: Transplantation into the dorsal skin with CS scaffold; Epi.fat: Epididymal fat pad

(A) Representative PLZF immunostaining of SSCs within seminiferous tubules. PLZF-positive cells are indicative of undifferentiated spermatogonia. Scale bar: 20 μm. (B) Quantitative analysis of PLZF-positive cells per tubule across experimental groups. a: Indicates a statistically significant difference compared to all other groups. b: Indicates a statistically significant difference compared to the Epi.fat group. Statistical significance was defined as *P*<0.05 (n = 3). PLZF: Promyelocytic leukemia zinc finger

## Conclusion

Allotransplantation of mouse ITT fragments embedded in CS scaffold into the epididymal fat pad and under the back skin areas improved spermatogonial survival and tissue integrity. Additionally, when comparing the two graft regions, the back skin area showed a better outcome.

Our study shows that using a CS scaffold for testicular tissue transplantation provides a model for future tissue engineering applications in treating male infertility.

## Data Availability

The data supporting the findings of this study are available upon request from the corresponding authors**.**

## Declaration

We have not used any AI tools or technologies to prepare this manuscript.
